# Sucrose intake lowers μ-opioid and dopamine D2/3 receptor availability in porcine brain

**DOI:** 10.1038/s41598-019-53430-9

**Published:** 2019-11-15

**Authors:** Michael Winterdahl, Ove Noer, Dariusz Orlowski, Anna C. Schacht, Steen Jakobsen, Aage K. O. Alstrup, Albert Gjedde, Anne M. Landau

**Affiliations:** 1 Department of Nuclear Medicine and PET Center, Aarhus University, Aarhus, Denmark; 2 Department of Neurosurgery and CENSE, Aarhus University Hospital, Aarhus, Denmark; 3 Department of Nuclear Medicine, University of Southern Denmark & Odense University Hospital, Odense, Denmark; 4 Department of Neuroscience, University of Copenhagen, Copenhagen, Denmark; 5 Department of Neurology and Neurosurgery, McGill University, Montreal, Canada; 6 Translational Neuropsychiatry Unit, Aarhus University, Aarhus, Denmark

**Keywords:** Feeding behaviour, Reward

## Abstract

Excessive sucrose consumption elicits addiction-like craving that may underpin the obesity epidemic. Opioids and dopamine mediate the rewarding effects of drugs of abuse, and of natural rewards from stimuli such as palatable food. We investigated the effects of sucrose using PET imaging with [^11^C]carfentanil (μ-opioid receptor agonist) and [^11^C]raclopride (dopamine D2/3 receptor antagonist) in seven female anesthetized Göttingen minipigs. We then gave minipigs access to sucrose solution for one hour on 12 consecutive days and performed imaging again 24 hours after the final sucrose access. In a smaller sample of five minipigs, we performed an additional [^11^C]carfentanil PET session after the first sucrose exposure. We calculated voxel-wise binding potentials (BP_ND_) using the cerebellum as a region of non-displaceable binding, analyzed differences with statistical non-parametric mapping, and performed a regional analysis. After 12 days of sucrose access, BP_ND_ of both tracers had declined significantly in striatum, nucleus accumbens, thalamus, amygdala, cingulate cortex and prefrontal cortex, consistent with down-regulation of receptor densities. After a single exposure to sucrose, we found decreased binding of [^11^C]carfentanil in nucleus accumbens and cingulate cortex, consistent with opioid release. The lower availability of opioid and dopamine receptors may explain the addictive potential associated with intake of sucrose.

## Introduction

Five percent of the world’s population are clinically obese^1^. As a hallmark of the metabolic syndrome, obesity is associated with type 2 diabetes, cardiovascular disease, respiratory problems, and risk of depression and possibly dementia^2^. The increased consumption of energy dense foods has exaggerated the physiologic distinction between homeostatic hunger that follows food deprivation, and hedonic hunger, or “craving”, which occurs in the absence of deprivation^3,4^. As the homeostatic regulation alone cannot account for the current rise in obesity, it is mandatory to test the effect on brain mechanisms of reward and pleasure of the addictive properties of highly palatable food.

Sucrose consumption is associated with obesity, and sucrose is increasingly considered an addictive substance^5^. Some findings are at variance with this claim due to difficulties in separating non-palatable food consumption from hedonic food responses, and in determining the addictive ingredient in processed food, as well as the different mechanisms by which food alters brain circuitry through natural pathways^6^. Nevertheless, in specific contexts, intake of sucrose does induce reward and craving, comparable in magnitude to those induced by addictive drugs, that lead to overconsumption and eventual obesity^6,7^.

Hunger is associated with “wanting” that is closely related to effects of dopaminergic neurotransmission in a number of reward circumstances^8^, but it remains unclear how the action of dopamine (DA) is modulated in response to compulsive eating. Consumption of palatable food is linked to “liking”, mediated primarily by the endogenous opioid system, especially the μ-opioid receptor (μOR)^9,10^, which can promote overconsumption when deregulated. In the present report, we test the claim that sucrose leads to opioid and dopamine release that lowers the availability of μOR and DA D2/3 receptors. The availability is an index of the number of unoccupied receptors available for tracer binding and in principle does not distinguish between ligand occupancy and receptor density^11^.

The onset of compulsive eating depends on multiple factors, and causal studies in humans raise ethical issues. The majority of studies therefore focus on feeding behavior in rats^12^. Although rats have a “sweet tooth”, their homeostatic mechanisms important to weight gain, metabolism, and type of fat accumulation, differ significantly from those of humans. The Göttingen minipig is a large omnivorous animal with a well-developed gyrencephalic brain, which can be imaged at sufficient resolution. Its well-defined subcortical and prefrontal cortical regions^13^ enable a more direct translation to human brain function. Here, we use positron emission tomography (PET) imaging to test *in vivo* μOR and DA D2/3 availability in a minipig model of subchronic sucrose exposure. In a smaller sample, we investigated the immediate effects on μOR occupancy after the first exposure to sucrose. Finally, we tested the relationship between the changes in receptor availability of the two tracers.

## Results

**Figure 1:**
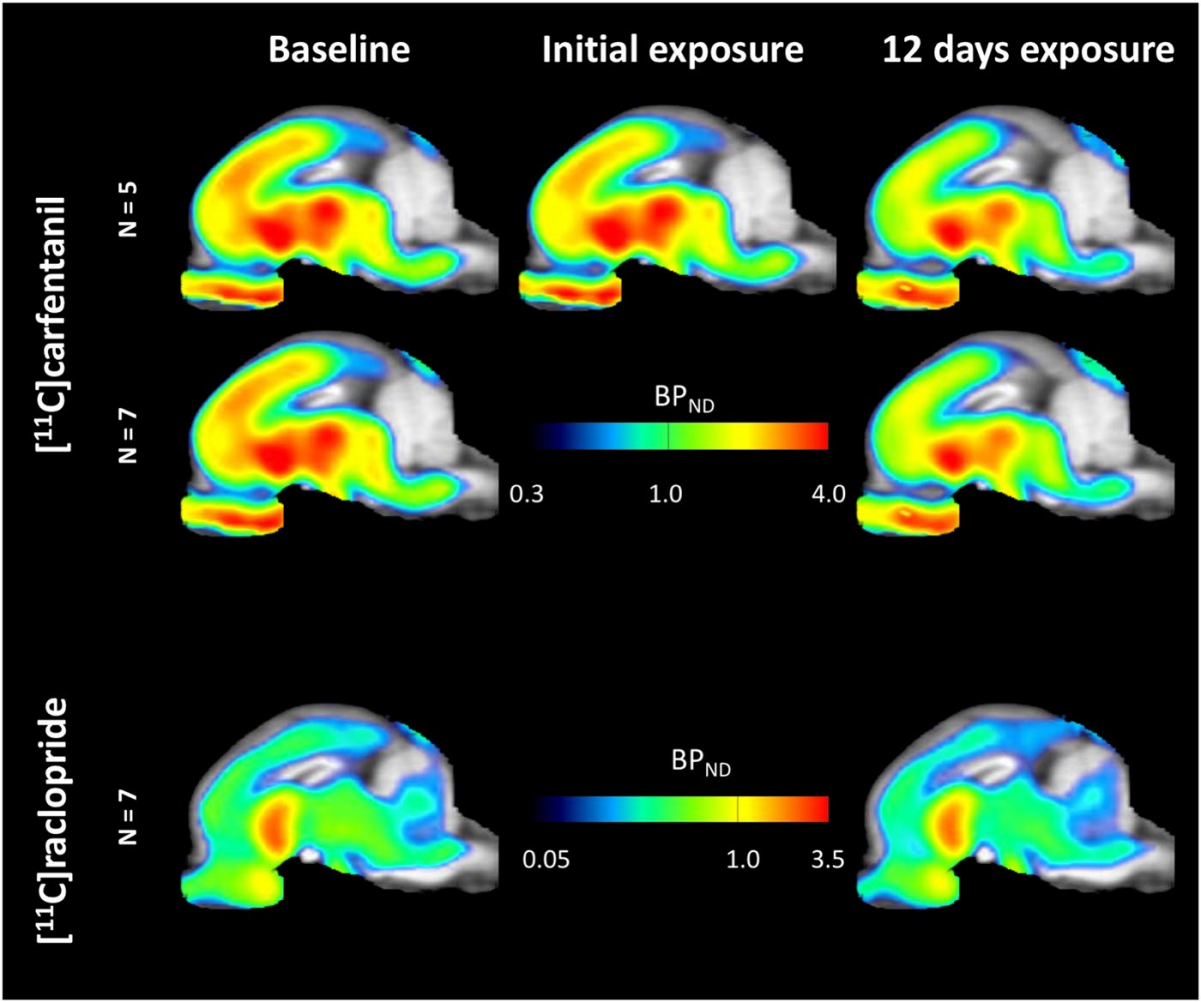
Average voxel-wise non-displaceable binding potential (BP_ND_) maps superimposed on MRI images in sagittal view. Data are presented for [^11^C]carfentanil BP_ND_ of the 5 minipigs imaged at baseline, after initial exposure to sucrose and after 12 days of sucrose exposure (top row). [^11^C]carfentanil BP_ND_ of all 7 minipigs imaged at baseline and after 12 days of sucrose access are presented in the middle row. [^11^C]raclopride BP_ND_ of all 7 minipigs imaged at baseline and after 12 days of sucrose access are shown in the bottom row. Note that the color scale is exponential to highlight the [^11^C]raclopride BP_ND_ in extrastriatal regions.

Average parametric maps of [^11^C]carfentanil and [^11^C]raclopride binding potential (BP_ND_) are shown in Fig. 1. To analyze the changes occurring after the first sucrose exposure in five minipigs compared to baseline, and one day after the 12^th^ sucrose access in seven minipigs compared to baseline, we used permutation theory and non-regionally restricted whole-brain analysis, the preferred method for samples of this size^14^.

### Initial sucrose exposure

**Figure 2:**
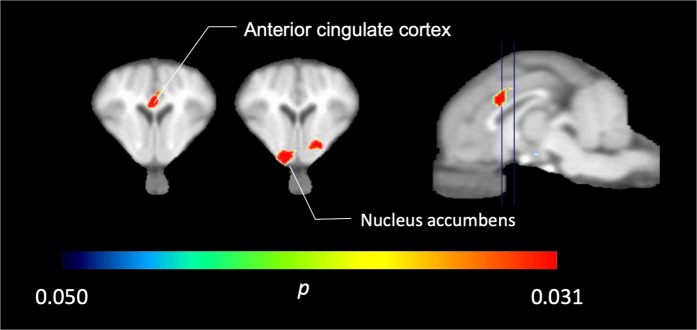
Significant decreases in [^11^C]carfentanil BP_ND_ after the first sucrose water exposure compared to baseline (n = 5). Only voxels with significant (*p* < 0.05) decreases are shown as colored areas projected onto T1 weighted MRI cuts at the level of the anterior cingulate cortex (left) and nucleus accumbens (middle) from a stereotaxic minipig brain atlas. Note that the maximum significance level achievable with 5 animals is 2^−5^ ≈ 0.031 (see color bar). Data are presented on coronal sections of the pig brain at the levels indicated on the sagittal image (right).

In the five minipigs imaged with [^11^C]carfentanil at baseline and immediately after the first sucrose exposure, we found significantly reduced tracer binding in the anterior cingulate cortex and the nucleus accumbens in response to sucrose, shown in color in Fig. 2, indicating p < 0.05. We detected as much as 14% decreased tracer binding in both areas compared to baseline.

### 12 days of sucrose access

**Figure 3:**
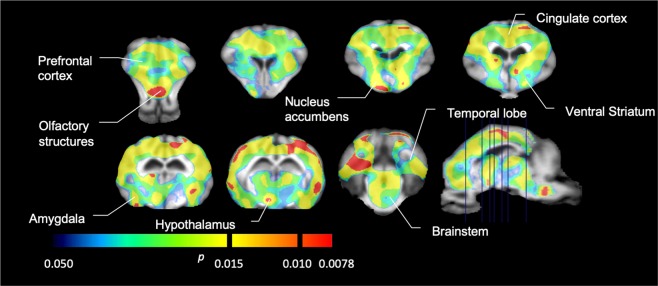
Significant decreases in [^11^C]carfentanil binding potential (BP_ND_) between baseline and after 12 days of sucrose water exposure (n = 7). The voxels with significant (*p* < 0.05) decreases are shown as colored areas projected onto T1 weighted MRI cuts from a stereotaxic minipig brain atlas. Data are presented on coronal brain sections at the levels indicated on the sagittal image (bottom right). Note that the maximum significance level achievable with 7 animals is 2^−7^ ≈ 0.0078 (see color bar).

**Figure 4:**
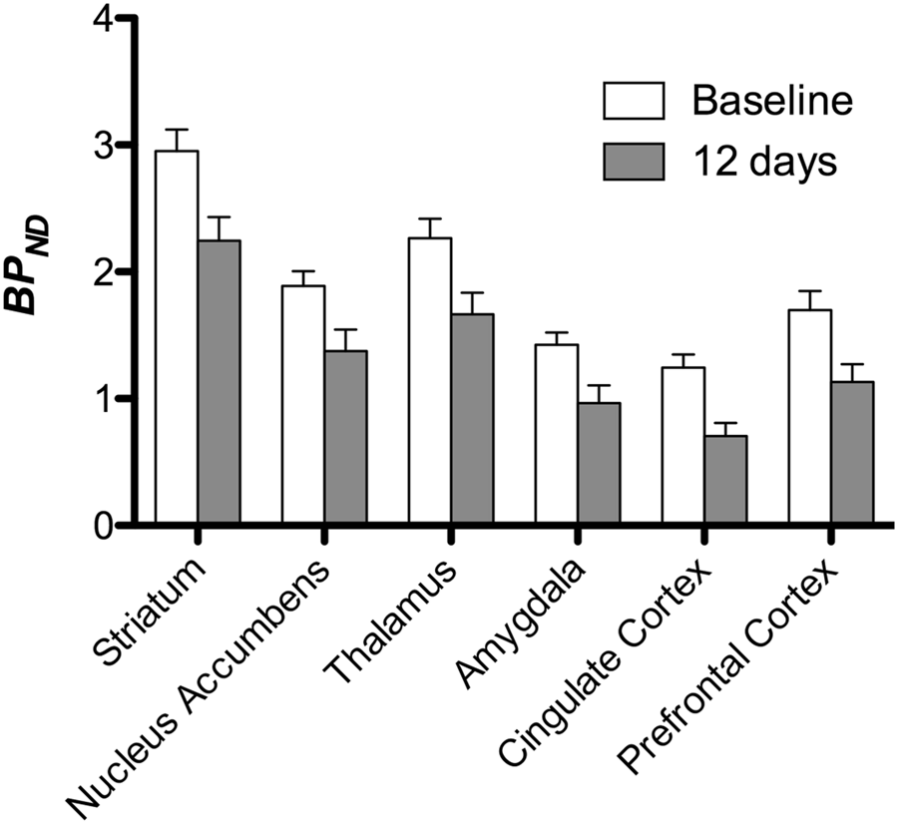
Regional analysis of [^11^C]carfentanil binding potential (BP_ND_) between baseline and after 12 days of sucrose water exposure (n = 7). Data are presented as means ± standard error.

We then performed the analysis of seven minipigs imaged with [^11^C]carfentanil at baseline and after 12 days of sucrose access and found significantly reduced tracer binding in sucrose-exposed animals compared to baseline. The most highly significantly affected regions are shown in red in Fig. 3 (p < 0.01) and include parts of the olfactory structures, nucleus accumbens/ventral striatum and the temporal cortex/lobe, followed by areas shown in yellow (p < 0.015) which included parts of the prefrontal cortex, cingulate cortex, amygdala and brainstem. In order to obtain BP_ND_ values and assess percent change, we performed regional analysis and obtained mean values in each region at baseline and after sucrose consumption (Fig. 4).

**Figure 5:**
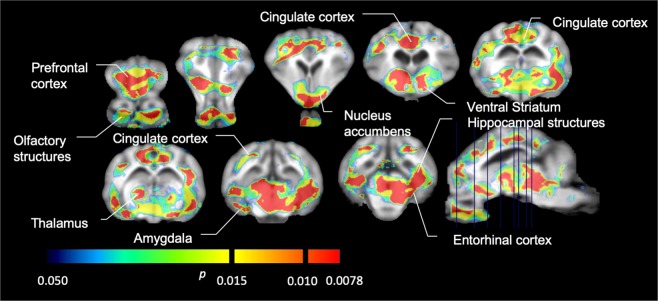
Significant decreases in [^11^C]raclopride binding potential (BP_ND_) between baseline and after 12 days of sucrose water exposure (n = 7). The voxels with significant (*p* < 0.05) decreases are shown as colored areas projected onto T1 weighted MRI cuts from a stereotaxic minipig brain atlas. Data are presented on coronal sections of the pig brain at the levels indicated on the sagittal image (bottom right). Note that the maximum significance level achievable with 7 animals is 2^−7^ ≈ 0.0078 (see color bar).

**Figure 6:**
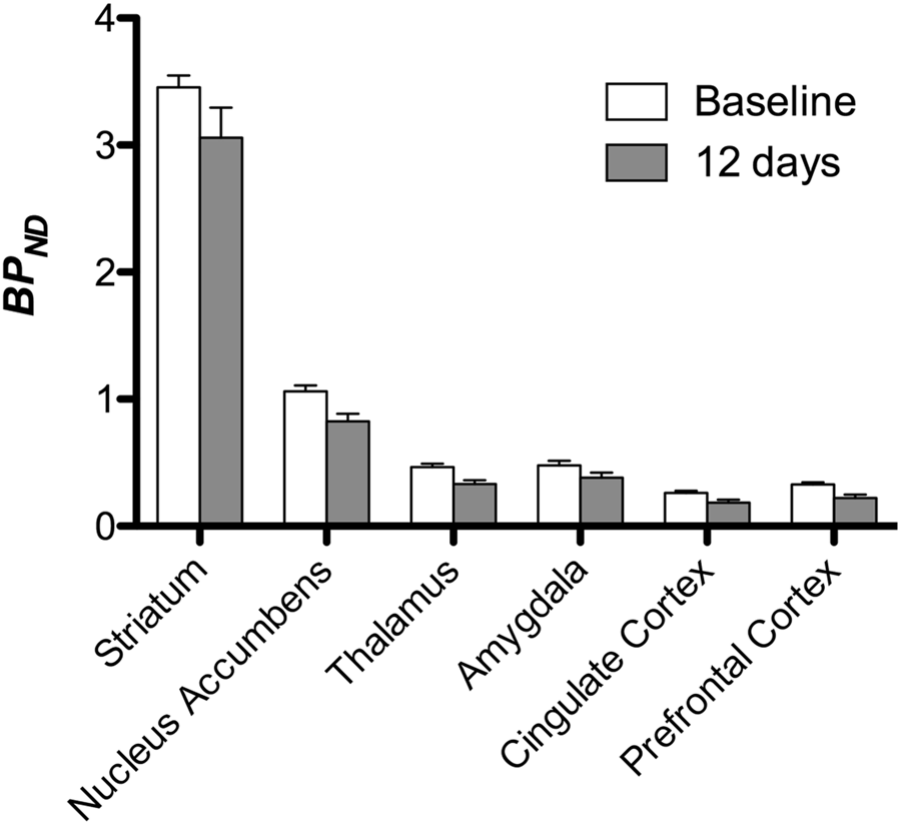
Regional analysis of [^11^C]raclopride binding potential (BP_ND_) between baseline and after 12 days of sucrose water exposure (n = 7). Data are presented as means ± standard error.

We used [^11^C]raclopride as the tracer of DA D2/3 receptors in striatal and extrastriatal brain regions in minipigs at baseline and after 12 days of sucrose access (Fig. 1). We found decreased tracer binding in sucrose-exposed animals, compared to baseline with largest effects (p < 0.01) in areas of the prefrontal cortex, nucleus accumbens/ventral striatum, cingulate cortex, amygdala, thalamus, mesencephalon, hippocampal regions, and olfactory areas (Fig. 5). Data from regional analysis are presented in Fig. 6.

### Correlations between [^11^C]raclopride and [^11^C]carfentanil data

**Figure 7:**
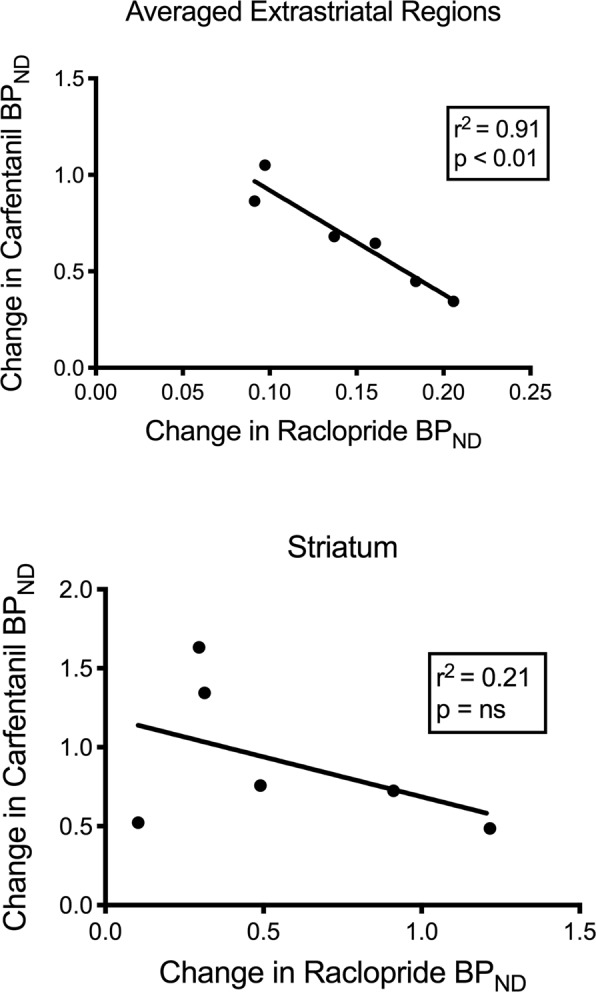
Correlations between pre- minus post- declines of [^11^C]raclopride and [^11^C]carfentanil binding potentials (BP_ND_) in minipigs with decreased tracer binding after sucrose intake (n = 6). Data from the averaged extrastriatal regions (top) and striatum (bottom) are presented. The coefficient of determination (r^2^) and the p values are shown for each graph.

We tested the potential correlation between [^11^C]raclopride and [^11^C]carfentanil values of BP_ND_ in striatal and non-striatal regions in minipigs at baseline and after 12 days of sucrose intake, with no associations observed. We then tested whether declines of tracer binding were correlated, and we compared the changes of BP_ND_ for [^11^C]raclopride with the changes of BP_ND_ for [^11^C]carfentanil only in the minipigs that had lower BP_ND_ of both tracers after sucrose intake (n = 6). We found significant negative correlations in averaged extrastriatal (r^2^ = 0.91, p < 0.01), but not in striatal, regions (Fig. 7).

## Discussion

We determined the effects of repeated intermittent access to sucrose on opioid and DA neurotransmission in mammalian brain. Longitudinal *in vivo* PET imaging of the μOR and DA D2/3 receptors revealed reduced receptor availability throughout the reward circuit, including the nucleus accumbens, prefrontal cortex, and the anterior cingulate cortex. The results clearly demonstrate that sucrose affects reward mechanisms in a manner similar to that of drugs of abuse.

The intake of sucrose as a palatable substance is known to release DA and induce dependency in rodents^15^, with sucrose shown to be even more pleasurable than cocaine in rodents in certain contexts. Thus, rodents work more intensely to obtain sucrose than cocaine, even in the absence of food deprivation^5^. However, the effects of sucrose are regulated both by the homeostatic system and by hedonic reward circuits^16,17^ that may mediate the distinction between nutritional and hedonic aspects of sucrose action^18^. We opted for a one-hour per day schedule in order to promote “binging”, as previous studies in rats had revealed a higher intake during the first hour of daily access in an intermittent schedule^15,19^. Behavioral studies of food intake often target food-restricted animals, but the design may not necessarily reflect the same neural mechanisms active in obesity. Pigs in the present study were not food restricted and were fed the usual amounts of their normal diet in addition to access to sucrose.

Opioid receptors (OR) are widely expressed in the brain, specifically in structures known to modulate eating and reward processes^20^. ORs have been shown to be important in the rewarding and relapsing effects of cocaine^21–24^. Alterations in binding have also been linked to the homeostatic responses to eating and the pleasure associated with palatable food^25^. In particular, “liking” of food is linked to the endogenous opioid system, especially the μOR^9,10^ in the shell of the nucleus accumbens and the ventral pallidum^26^. Infusions of a μOR agonist into distinct portions of the nucleus accumbens and ventral pallidum strongly enhance “liking” behaviors, including tongue protrusions and paw licking, following increased palatable intake of food^27–29^. Further evidence for opioid signaling in the processing of hedonic regulation comes from μOR antagonists that attenuate consumption of palatable chow in both *ad libitum*-fed and food restricted animals, but with a more limited effect on the intake of non-palatable standard pellets^30,31^. In humans, μOR antagonists decrease short-term food intake and reduce pleasantness of palatable foods^32–34^. Opioid signaling in the basolateral amygdala also contributes to food “wanting” through modulation of reward seeking and the incentive value of food^35^.

With [^11^C]carfentanil, we obtained images of tracer binding that is sensitive both to μOR levels and to the brain’s release of endogenous opioids^36,37^. We detected immediate loss of μOR availability in areas of the nucleus accumbens and anterior cingulate cortex, specific brain regions of the reward pathway, after initial consumption of sucrose by five minipigs, consistent with endogenous opioid release. Previous studies have shown that palatable food can lead to feelings of pleasure^38^ by stimulating opioid release. After 12 days of sucrose access, we observed decreased [^11^C]carfentanil binding, which has several possible explanations^39^ including endogenous opioid release and binding to μOR, μOR internalization as a result of increased opioid binding, and increased DA D2/3 receptor activation leading to heterologous desensitization of μOR^40^.

In support of the present findings, [^11^C]carfentanil studies of patients with bulimia^41^, obesity^42–44^, and binge-eating disorder^45^, show decreased receptor availability. However, these are chronic conditions whereas the minipigs only received sucrose for 12 days. In a study of acute feeding behavior in healthy men, feeding led to robust and widespread endogenous cerebral opioid release, both in the presence and absence of hedonia, suggesting that opioid release reflects metabolic and homeostatic, as well as hedonic, responses^25^. This study, together with another that imaged patients after a chocolate-flavored liquid meal^44^, is directly relevant to the acute study of five minipigs after the first sucrose exposure, but is different from the subchronic sucrose-exposure study over 12 days where the reduced receptor availability more likely reflects repeated overstimulation and concomitant downregulation of μOR.

The prefrontal cortex is important in decision-making and ascribing value to items and therefore the μOR in the prefrontal cortex may be accountable for the altered evaluation of food saliency, which can raise the addictive potential of food. We have found decreased binding in the prefrontal cortex, consistent with previous studies showing that high fat diet reduces levels of μOR mRNA in the prefrontal cortex^46^ and that infusion of a μOR agonist in the prefrontal cortex increases intake of sweet food^47^. Again, however, the issue arises whether the high fat diet is a more chronic condition that more likely mediates receptor down-regulation, compared to the shorter-term sucrose-feeding design, suggesting sustained release of endogenous opioids that displaces tracer carfentanil bound to μOR, even after 12 days of sucrose.

DA has been implicated in rewards both from drugs and behavior. Chronic cocaine use has been found to inhibit DA signaling^48^. DA D1 and D2/3 receptor levels are altered by nicotine in pig brain^49^, and in non-human primates with a history of cocaine abuse^50^, consistent with the downregulation of D2/3 receptors in the brains of human cocaine addicts^51,52^. As for drugs of abuse, sucrose has been shown to upregulate DA D1 receptors^19^ and increase DA release^53^, reinforcing the role of DA in “wanting” in relation to palatable food. Previous PET studies have demonstrated a decrease in striatal DA D2/3 receptor availability in morbid obesity vs average weight^54,55^, similar in magnitude to the reduction in drug-addicted patients^56^, and in animal with models of obesity^57^. In rodent studies, D2/3 receptor knockdown in the striatum promotes the development of compulsive food seeking in rats with access to palatable food^57^.

Our observations of decreased D2/3 receptor availability of the pig may indicate increased DA levels in response to the incentive salience associated with the sucrose intake since DA is released as part of the wanting of drugs of abuse and other pleasurable activities^52,58–60^. As the pigs were anesthetized during the imaging, and had not received sucrose in 24 hours, the decreased D2/3 BP_ND_ more likely reflects a reduction in the number of receptors in response to prolonged increase of DA release at each of the 12 days of sucrose access. The reduction can raise brain reward thresholds, associated with down-regulation of striatal DA D2 receptors. This may explain the increased susceptibility to drugs of abuse seen in previous studies of rats overeating sucrose that led to cross-sensitization to cocaine, hyperactivity after low dose amphetamine, increased alcohol intake when abstaining from sucrose, and tolerance to the analgesic effects of opiates^6^.

A previous study of obesity in the Göttingen minipig identified decreased cerebral blood flow in the nucleus accumbens, ventral tegmental area (VTA) and prefrontal cortex, with single photon emission computed tomography (SPECT) of brain^61^. Consistent with these findings, we observed reduced DA D2/3 binding in the ventroforebrain region containing the nucleus accumbens and in the prefrontal cortex. Extracellular levels of DA are increased 3-fold in the nucleus accumbens after sucrose intake in freely-moving rats undergoing microdialysis^62^. In sucrose dependent animals, repeated sucrose intake can lead to release of DA from the shell of nucleus accumbens^63^. Animals fed a restricted diet with limited access to sucrose had lower DA D2 receptor binding in the nucleus accumbens shell and the dorsal striatum^64^. Restricted high fat and sucrose diets can lead to sustained downregulation of D1 and D2 receptor mRNA in the nucleus accumbens^65^. A microdialysis study of the effects of palatable food revealed increased DA release in the nucleus accumbens and prefrontal cortex when the food was still considered novel; once the rats were accustomed to the new food, the increased release was blunted in the nucleus accumbens, but not in the prefrontal cortex^66^. The differential susceptibility to habituation and conditioning of the activity in two regions may explain the larger increase observed in prefrontal cortex than in nucleus accumbens of minipigs exposed to the same palatable substance that lost novelty after twelve days. However, as we did not image minipigs with [^11^C]raclopride after the first sucrose administration, this explanation is speculative.

The prefrontal cortex modulates executive function, decision-making, and self-control^67^. Dysfunctional DA neurotransmission in the prefrontal cortex impairs modulation of reward processing, suggesting impaired executive function and decision-making skills in obese individuals^68,69^. Moreover, a human PET study correlated decreased frontal cortex metabolism with decreased striatal D2 binding in obesity^70^. Here, we find reduced D2/3 receptor availability in the prefrontal cortex including the orbitofrontal cortex of pigs exposed to the sucrose regimen.

Dopaminergic neurons of the VTA send projections to the hippocampus and amygdala, where they support habit-like behaviors^71^ and mediate the encoding and retrieval of conditioning to drug^72,73^ and food cues^74,75^. Human brain imaging has shown hippocampal activation in response to food craving and tasting^76^. Consistent with our findings of a reduced hippocampal and amygdalar D2/3 receptor availability in response to sucrose, human brain mapping with [^18^F]fallypride showed cocaine cue-induced DA release in amygdala and hippocampus^77^. In rodent brains, cocaine cue exposure triggered DA release in the amygdala^78^, and alterations of amygdala DA levels influenced cue-induced cocaine-seeking behavior^79^.

In a study of obese individuals, the association between D2/3 and μOR availabilities, known to exist in striatal regions of lean individuals, was disrupted in the ventral striatum^80^. We compared the values of BP_ND_ of the two tracers to test if the data reproduced this effect. Unlike lean humans, the present brains of pigs had no correlation between the values of BP_ND_ of the two tracers, at baseline or after the exposure to sucrose. We then tested whether the animals with the largest declines of tracer raclopride binding would also have the largest decreases of tracer carfentanil binding, but instead we found a negative correlation in the averaged extrastriatal regions, suggesting that animals with the greatest change of the binding potential of tracer raclopride had the lowest change of the binding potential of tracer carfentanil. The inverse relation between the changes suggests that the effects of sucrose intake on the availabilities of the respective receptors are regulated in opposite directions. It is known that excessive consumption of palatable food, or drugs, can be driven by wanting or liking, or both^60,81^. It is possible that the magnitude of wanting driven by dopamine negates the magnitude of liking driven by opioids, or vice versa. Recent evidence points to roles of GABA_A_ receptors in the VTA and cholinergic terminals in striatum and possibly cortex that act as switches between dopamine-dependent and dopamine-independent mechanisms of opioid action^82,83^ that may explain the reciprocity of dopamine and opioid effects in porcine extrastriatal regions determined here.

A shortcoming of PET, also in comparatively large animals, is the limited spatial resolution of the tomography that affects the results from small brain regions involved in food-associated behaviors. However, despite these concerns, [^11^C]raclopride binding previously was recorded both in striatal and extrastriatal regions^84–87^. The use of [^11^C]raclopride to label the same type of receptors raises no concern about potential affinity differences that may affect the use of separate tracers for the same receptors in different regions. Recent studies included records of extrastriatal binding of [^11^C]raclopride. Alakurtti *et al*. found good reproducibility of measures of striatal raclopride binding in the striatum, with only good to moderate reproducibility in the cortex^85^. In a later study, Svensson *et al*. discussed several issues affecting the use of [^11^C]raclopride as a marker of extrastriatal D2/3 receptors in a study of healthy humans, including poor reproducibility in cortex and limited decline of extrastriatal binding in frontal cortex in response to a D2/3 blocking agent^88^. The test-retest comparisons revealed variabilities of 4–7% in striatum and 13–59% in cortical regions, but the time between examinations averaged 20 days, unlike the more informative 1–2 days of most studies. A number of factors in the lives of those subjects may have had time to influence the findings. Indeed, we show here that merely adding sucrose consumption to a morning routine for 12 days may have influenced binding measures obtained two weeks later. Other factors as common as playing video games, shopping, entering new romantic relationships and sexual activity, using drugs or changing diet and exercise may influence extrastriatal dopamine levels with potential for great variation of datasets. The current study in minipigs introduced a well-controlled set-up with the only variable being the absence or presence of sucrose in the diet. In this context, the data from seven animals had sufficiently low variability in relevant extrastriatal regions to identify a statistically significant reduction of binding in response to sucrose.

A limitation of the current study is the use of anaesthetics required to ensure immobility during *in vivo* imaging of animals. The effects of specific anaesthetics, and their interactions with drugs or other interventions, can confound the binding of radioligands^89,90^. Ketamine is an anti-glutamatergic drug with rapid antidepressant effects in sub-anaesthetic doses^91–93^, that do not reduce striatal [^11^C]raclopride binding in humans^94^. However, S-ketamine was found to reduce binding availability of dopamine D2/3 receptors in striatum of conscious non-human primates^95^. Isoflurane is a common anaesthetic in animal PET. In previous studies, we found striatal accumulation of [^11^C]SCH23390, a radioligand of the dopamine D1 receptors to be significantly higher in minipigs anesthetized with isoflurane rather than propofol, suggesting susceptibility of the dopaminergic neurotransmission to effects of anaesthesia^96^. In the current study, all minipigs were imaged at both timepoints under ketamine pre-medication and isoflurane anaesthesia, making the present comparisons valid.

## Conclusion

Excessive consumption of palatable food may both cause, and become the result of, addiction with direct consequences to health by obesity. We tested the claim that opioids and dopamine mediate rewards, important to survival as well as to abuse of drugs. Minipigs with intermittent access to a sucrose solution on 12 consecutive days demonstrated decreased dopamine D2/3 and μ−opioid receptor availability in striatal and extrastriatal brain regions, implying that foods high in sucrose influence brain reward circuitry in ways similar to those observed when addictive drugs are consumed. Initial single exposure to sucrose was consistent with opioid release in brain regions active in reward. The changes of opioid and dopamine availability explain the addictive potential of sucrose consumed in excess.

## Materials and Methods

### Animal ethics

This study was approved and regulated by the Danish Animal Experiments Inspectorate and all experiments were carried out in accordance with the 2010/63/EU directive of the European Parliament and of the Council on the Protection of Animals Used for Scientific Purposes and the ARRIVE guidelines. We used seven fourteen-month old female Göttingen minipigs (Ellegaard, Dalmose, Denmark). Minipigs were fed a pellet diet (6 dL, 2 times daily, Special Diets Services, Aarhus, Denmark) with tap-water available *ad libitum*. The environmental temperature was 20–22 °C, relative humidity 50–55%, and air was changed eight times every hour.

### Intermittent sucrose consumption

We imaged seven minipigs with [^11^C]raclopride and [^11^C]carfentanil at baseline, and again one day after 12 consecutive days of sucrose water exposure. Sucrose exposure consisted of one hour of sucrose (sucrose, Dansukker, Copenhagen, Denmark) water access (500 grams of sucrose in 2 liters of water), daily during a 12-day period. The amount of sucrose intake was recorded and all minipigs consumed 2 liters on each day. We also imaged five of the same minipigs with [^11^C]carfentanil, 30 minutes after the first sucrose access, in order to study acute opioid release.

The minipigs gained an average of 13.6% body weight from 25.4 kg (±0.73 SEM) at baseline to 28.9 kg (±0.69 SEM) after the 12-day sucrose exposure, which was significantly higher (one-tailed t-test, p < 0.001) than the increases observed in a sample of control minipigs obtained in previous studies, where weights increased on average by only 4.9%, during the same developmental period.

### Brain PET Imaging

We fasted pigs overnight with free access to water prior to imaging. We pre-medicated and anesthetized minipigs as described previously^97^ and placed them supine in a PET/CT device (Siemens Biograph 64 Truepoint PET). We performed a low-dose CT scan prior to each PET acquisition for anatomical definition and attenuation correction of PET emission data. We intravenously administered [^11^C]raclopride at baseline (360 ± 18 MBq, specific activity 77 ± 76 GBq/μmol, injected mass 0.12 ± 0.08 μg/kg) and after 12 days of sucrose (374 ± 54 MBq, specific activity 127 ± 85 GBq/μmol, injected mass 0.06 ± 0.05 μg/kg), and [^11^C]carfentanil at baseline (377 ± 43 MBq, specific activity 311 ± 195 GBq/μmol, injected mass 0.03 ± 0.02 μg/kg) and after 12 days of sucrose (337 ± 71 MBq, specific activity 177 ± 157 GBq/μmol, injected mass 0.06 ± 0.08 μg/kg) via ear vein, in 10 mL saline, during the first minute of a 90-minute scan. We reconstructed PET data using TrueX 3D OSEM (3 iterations, 21 subsets), a 256 × 256 × 109 matrix, and a 2-mm Gauss filter, using a time-frame structure of 5 × 60, 3 × 300, 4 × 600, 2 × 900 seconds (total 14 frames, 90 minutes). At baseline and after 12 days of sucrose, minipigs were imaged with both tracers injected at least 100 minutes apart, due to the half-life of [^11^C] PET tracers. Upon completion of the final PET session, we euthanized minipigs under deep anesthesia by an intravenous overdose of pentobarbital (100 mg/kg).

### Quantitative analyses and statistics

We performed preprocessing steps using PMOD 3.7 (PMOD Technologies Ltd, Zurich, Switzerland). To define the stereotactic transformation parameters from time-averaged PET images, we used ligand-specific templates. We applied the generated transformation matrices and warping fields onto the corresponding dynamic PET time series. We generated parametric images of [^11^C]raclopride binding potential (BP_ND_) by means of the multilinear reference tissue method of Ichise and co-workers^98^. We created a custom-made mask of the cerebellum that excluded the vermis to obtain the cerebellar tissue radioactivity over time in a region of negligible DA D2/3 receptor density. We generated parametric images of [^11^C]carfentanil using an implementation of the Logan reference tissue model^99,100^ with t* = 30 min. Studies of [^11^C]carfentanil binding in human brain have used the occipital cortex as a reference region^36^; however, in the pig, according to the time activity curves, non-displaceable binding was lower in the cerebellum than in the occipital cortex, consistent with findings from a rat autoradiography study^101^. We therefore selected the cerebellum as the reference region in the current study.

### Statistical analysis

We subjected maps to a voxel-wise analysis with Statistical Non-Parametric Mapping (SnPM v13.01, http://warwick.ac.uk/snpm) SPM toolbox that utilizes non-parametric permutation theory to provide a framework for statistical inference, an approach shown to work well for small samples due to strict control of false positives^14^ and applied as previously described^102^. An expert in pig neuroanatomy (DO) compared the resulting images thresholded to 5% significance level to a high-resolution Göttingen minipig atlas^103,104^ to define and label regions of decreased DA D2/3 and μOR BP_ND_ from baseline to the post-sucrose condition. We then performed a region-of-interest (ROI) analysis in order to extract BP_ND_ values of specific regions found to be of interest based on the SnPM analysis, including the striatum, nucleus accumbens, thalamus, amygdala, cingulate cortex and prefrontal cortex. No additional statistics were performed on the ROI analysis, since these regions were already found to be significant using SnPM.
